# Refractory Cardiogenic Shock Secondary to Acute Myocarditis in a Child with Multisystem Inflammatory Syndrome Associated with COVID-19: A Case Report

**DOI:** 10.31729/jnma.6376

**Published:** 2022-11-30

**Authors:** Susmin Karki, Saurav Agrawal, Aadesh Rayamajhi, Asmita Parajuli, Sushil Kumar Yadav, Srijana Basnet

**Affiliations:** 1 Maharajgunj Medical Campus, Tribhuvan University Teaching Hospital, Maharajgunj, Kathmandu, Nepal; 2 Department of Paediatrics, Tribhuvan University Teaching Hospital, Maharajgunj, Kathmandu, Nepal

**Keywords:** *cardiogenic shock*, *COVID-19*, *kawasaki disease*, *myocarditis*, *Nepal*

## Abstract

Children with Coronavirus disease 2019 infection usually have mild symptoms but rarely may present with a life-threatening condition called a multisystem inflammatory syndrome. We report a case of COVID-19-related multisystem inflammatory syndrome in an 8-year-old boy who presented with cardiogenic shock due to acute myocarditis with no features of Kawasaki disease. Cardiogenic shock was refractory to fluids and inotropes. Later, this case was successfully managed with hydrocortisone and intravenous immunoglobulin. Therefore, this case report highlights keeping a lookout for such atypical presentations and early referral to a higher center for timely intervention and aggressive therapy specifically directed against the underlying inflammatory process to ameliorate the outcomes.

## INTRODUCTION

Coronavirus disease 2019 (COVID-19) is usually a mild disease in children, therefore primary infection may go unnoticed and sometimes lead to rare complications after 1-6 weeks following SARS-CoV-2 infection, referred to as Multisystem Inflammatory Syndrome (MIS-C). The incidence of MIS-C is 316 persons per 1,000,000 SARS-C0V-2 infections.^1^ The Centers for Disease Control and Prevention (CDC) and the World Health Organization (WHO) has provided a case definition.^2,3^ MIS-C presenting with symptomatic myocarditis and refractory cardiogenic shock is rarely reported.

## CASE REPORT

A previously healthy 8-year-old boy presented to the emergency department with chief complaints of fever for 7 days, abdominal pain for 3 days and difficulty in breathing for 1 day. There was no history of contact with a COVID-19 patient or symptomatic acquaintances. His initial vitals included temperature (38.4°C), heart rate (148 beats/min), respiratory rate (84 breaths/min), blood pressure (90 systolic/80 diastolic mm of hg), SpO_2_ (94% with face mask), capillary refill time (<2 sec). There was no significant family history.

Physical examination revealed a well-nourished boy appeared to be lethargic, ill-looking, pale and oriented to time, place and person. On per abdomen examination, there was generalized abdominal tenderness, moderate distension and tender hepatomegaly. There was no significant history of lymphadenopathy, strawberry tongue, cracked lips, conjunctivitis, swelling of hands and feet or rashes on the skin. The findings of other systems were unremarkable.

On the 1^st^ day of hospitalization, nasopharyngeal swabs were negative for SARS CoV-2 or Influenza (A and B) ribonucleic acid. Due to increasing respiratory distress and cardiogenic shock, the patient was transferred to the pediatric intensive care unit (PICU). The child was kept on mechanical ventilation during the initial 6 days of hospital stay and fluid and inotropes were started (dopamine 15 mcg/kg/min, dobutamine 15 mcg/kg/min and adrenaline 0.4 mcg/kg/min) but the child's hemodynamic status did not improve. Therefore, hydrocortisone 150 mg/day was administered for the management of refractory shock.

**Figure 1:**
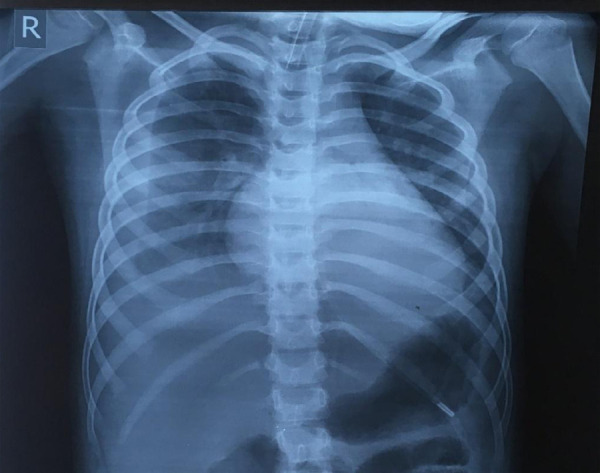
Chest x-ray shows pleural effusion in both the right and left lungs.

Laboratory tests showed anemia (Hb-9.5 g/dl, hematocrit-28.3%), 16,820/mm^3^ leucocyte (81% polymorphonuclear cell) with only 11% lymphocytes. There was increase in value of inflammatory markers and enzymes, such as C-reactive protein (CRP) (117 mg/L) and D-dimer (9.62 mcg/ml), ferritin (1650 ng/mL), fibrinogen (650 mg/dL), aspartate aminotransferase (55 U/L), lactate dehydrogenase (467 U/L) with hypoalbuminemia (2.7 g/dL). Other parameters such as prothrombin time, activated plasma thromboplastin time, lactic acid, creatinine, alanine aminotransferase, creatinine phosphokinase, and troponin I were within normal limits. Blood gas analysis showed a pH of 7.41, PCO_2_ of 37 mm Hg, and PO_2_ of 70 mm Hg. Chest x-ray showed bilateral pleural effusion (Figure 1), and ultrasonography of the abdomen-pelvis showed a small amount of free fluid in the abdominal cavity.

**Figure 2:**
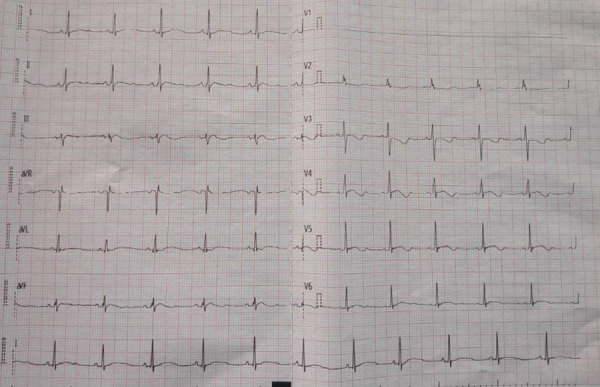
Electrocardiogram showing sinus tachycardia with inversion of T wave in all chest leads.

An echocardiogram done on the 5^th^ day of admission showed global left ventricular wall hypokinesis, reduced left ventricular ejection fraction (25-30%), mild mitral regurgitation and tricuspid regurgitation, with minimal pericardial effusion with no clot or vegetation. Antistreptolysin O titre was normal. Blood culture was sent, and the patient was treated with empirical antibiotics such as vancomycin 2.4 g/day, chloramphenicol 2 g/day and meropenem 2.25 g/day for 7 days with suspicion of sepsis. Investigations such as latex agglutination test for evidence of Salmonella typhi and paratyphi, indirect immunofluorescence test for Immunoglobulin M (IgM) and Immunoglobulin G (IgG) against Orientia tsutsugamushi, dengue viruses, Leptospira interrogans which was endemic to patient's residence were negative. Electrocardiogram revealed sinus tachycardia with inversion of T wave in all chest leads (Figure 2).

The clinical features, echocardiography and electrocardiogram findings were suggestive of myocarditis and medication such as dobutamine 15 mcg/kg/min, furosemide 30 mg/day, enalapril 2.5 mg/day and spironolactone 25 mg/day were administered. With haemoglobin of 9 g/dL and haematocrit of 28%, he received red cell concentrate. On the day 4 of hospital stay with a negative blood culture report, a test for SARS-CoV-2 IgG antibody was sent, and the test result was positive. He met the diagnostic criteria of a multisystem inflammatory syndrome in children as per the CDC and the WHO.^2,3^ Therefore, Intravenous immunoglobulin (IVIG) (50 gm) was given over 24 hours. Anticoagulation with enoxaparin 30 mg/day, aspirin 75 mg/day and methylprednisolone 800 mg infused over 1 hour was started. The child gradually improved and was discharged on the 11^th^ day of hospital admission. The child was doing well during follow-up visits with normal laboratory parameters and radiological findings. Repeat echocardiography after the 2^nd^ and 6^th^ week of illness was normal. Enoxaparin was continued for 2 weeks and aspirin for 6 weeks after discharge.

## DISCUSSION

We report a case of MIS-C in a child treated in our tertiary care center. This case had cardiogenic shock due to acute myocarditis which was refractory to both fluid and inotropes. This condition is being recently recognized in Nepal; however, there is a lack of published reports from Nepal. Our patient met both CDC and WHO criteria for MIS-C.^2,3^ We believe this case report offers several important lessons regarding this novel disease.

A systematic review and meta-analysis done in Iran on MIS-C-related clinical presentation reported a majority of children with fever, and mucocutaneous involvement resembling atypical Kawasaki and multiorgan failure.^4^ Our patient did not fulfill the criteria for Kawasaki disease and an echocardiogram done twice at an interval of 14 days revealed normal coronary arteries so atypical Kawasaki disease was ruled out from diagnosis. Our case presented with fever, abdominal pain, and difficulty in breathing and eventually landed into cardiogenic shock after one day of hospitalization.

There was no history of contact with a COVID-19 patient or symptomatic acquaintances and his Reverse transcription-Polymerase Chain Reaction (RT-PCR) was negative. Since he presented to us when COVID-19 was endemic in our part of the world therefore after excluding other common illnesses manifesting in a similar pattern, serology for COVID-19 was sent which was positive. Hence, this case highlights the importance of keeping a high index of suspicion of this condition when children have a persistent fever with features of shock, especially during this pandemic situation.

To date, limited awareness has been afforded to possible SARS-CoV-2-related cardiovascular injury in the pediatric population. The diagnosis is presumed on clinical presentation and noninvasive diagnostic methods such as cardiovascular magnetic resonance imaging (MRI).^5^ Cardiac MRI was not performed because of the cost factor and endomyocardial biopsy is not practiced in our setting.^6^ In our case myocarditis was diagnosed based on clinical findings, echocardiography and ECG report.^7^ While myocardial involvement was severe, there was no significant valvulitis with normal Antistreptolysin O titre further arguing against acute rheumatic fever which is the most common cause of acquired heart disease in our part of the world. Therefore, those diagnoses were subsequently ruled out. Nonspecific serum markers of inflammation including leukocytes and C-reactive protein were elevated in our case suggestive of acute myocarditis, but normal values do not exclude an acute myocardial inflammatory process. The clinical presentation of myocarditis is variable ranging from asymptomatic to devastating illness with cardiogenic shock.^5^

The basic treatment measures of cardiogenic shock include fluid resuscitation to obtain euvolemia, vasopressors, and inotropes to which our patient was refractory.^8^ Treating MISC patients with immunoglobulin and methylprednisolone effectively restores left ventricular systolic function.^9,10^ A similar improvement in left ventricular ejection fraction (65%) was observed in our case after one day of administration of such medications. The SARS-CoV-2 virus might have involved myocardial tissue through an immune-inflammatory process eventually leading to cardiogenic shock but due to lack of direct evidence, we assume acute myocarditis in our case is associated with COVID-19, but not a complication.

MISC should be suspected in a child with persistent fever with clinical features of myocarditis and refractory cardiogenic shock. Relevant investigation as per CDC/WHO diagnostic criteria helps to establish the diagnosis. Children with MIS-C requiring hospital admission should undergo a cardiac workup and close cardiovascular monitoring to identify and treat timely life-threatening cardiac complications. Timely intervention and aggressive therapy specifically directed against underlying inflammation ameliorate the outcome.
